# OPERATIONALIZING THE FRAILTY INDEX BASED ON WEARABLE SENSOR TO ASSESS FUNCTIONAL PERFORMANCE IN OLDER ADULTS

**DOI:** 10.1093/geroni/igz038.2513

**Published:** 2019-11-08

**Authors:** Hung Nguyen, Jacqueline Yang, Mohsen Zahiri, Bijan Najafi

**Affiliations:** Baylor College of Medicine, Houston, Texas, United States

## Abstract

Frailty status is a well-known predictor of adverse health outcomes and functional performance. An assessment tool based on a wearable sensor was developed to quickly assess frailty using an upper extremity flexion and extension test. However, the current tool has relied on conventional frailty assessment to classify the frailty status of the participant. The aim of this study is to operationalize the frailty index based on wearable sensor to classify frailty status of older adults. 104 older adults were recruited for the study (age=78.6 ±9.7 years old). Participants were asked to perform a quick 20-second upper flexion and extension task while wearing a gyroscope on the wrist. A sensor-based frailty index (FI) was derived using parameters extracted from the sensor. Participants were also assessed using the Fried Phenotype Criteria (FC) and were classified into three groups: robust, pre-frail, and frail. Mean-shift clustering algorithm was used to operationalize the FI by identifying the cut-off point for each group. Grip strength and physical activity level were used as functional outcome measures. Regression analysis (r) was used to compare the correlation of the FC and FI with the identified metrics. Bivariate analysis show that grip strength was highly associated with the sensor-based frailty classification (r=-0.547) and FC (r =-0.503). The sensor-based classification was significantly associated with walking activity (r=-0.355). The results showed that the sensor-based frailty assessment tool could be used to quickly classify frailty status in older adults and eliminated the need for subjective and time-consuming evaluation.

